# Modulation of Glucose Transporter 1 (GLUT1) Expression Levels Alters Mouse Mammary Tumor Cell Growth In Vitro and In Vivo

**DOI:** 10.1371/journal.pone.0023205

**Published:** 2011-08-03

**Authors:** Christian D. Young, Andrew S. Lewis, Michael C. Rudolph, Marisa D. Ruehle, Matthew R. Jackman, Ui J. Yun, Olesya Ilkun, Renata Pereira, E. Dale Abel, Steven M. Anderson

**Affiliations:** 1 Department of Pathology, University of Colorado School of Medicine, Anshutz Medical Campus, Aurora, Colorado, United States of America; 2 Department of Medicine, Division of Endocrinology, Metabolism, and Diabetes, University of Colorado School of Medicine, Anshutz Medical Campus, Aurora, Colorado, United States of America; 3 Division of Endocrinology, Metabolism, and Diabetes Program in Molecular Medicine, School of Medicine, University of Utah, Salt Lake City, Utah, United States of America; Huntsman Cancer Institute, University of Utah, United States of America

## Abstract

Tumor cells exhibit an altered metabolism characterized by elevated aerobic glycolysis and lactate secretion which is supported by an increase in glucose transport and consumption. We hypothesized that reducing or eliminating the expression of the most prominently expressed glucose transporter(s) would decrease the amount of glucose available to breast cancer cells thereby decreasing their metabolic capacity and proliferative potential.

Of the 12 GLUT family glucose transporters expressed in mice, GLUT1 was the most abundantly expressed at the RNA level in the mouse mammary tumors from MMTV-c-ErbB2 mice and cell lines examined. Reducing GLUT1 expression in mouse mammary tumor cell lines using shRNA or Cre/Lox technology reduced glucose transport, glucose consumption, lactate secretion and lipid synthesis *in vitro* without altering the concentration of ATP, as well as reduced growth on plastic and in soft agar. The growth of tumor cells with reduced GLUT1 expression was impaired when transplanted into the mammary fat pad of athymic nude mice *in vivo*. Overexpression of GLUT1 in a cell line with low levels of endogenous GLUT1 increased glucose transport *in vitro* and enhanced growth in nude mice *in vivo* as compared to the control cells with very low levels of GLUT1.

These studies demonstrate that GLUT1 is the major glucose transporter in mouse mammary carcinoma models overexpressing ErbB2 or PyVMT and that modulation of the level of GLUT1 has an effect upon the growth of mouse mammary tumor cell lines *in vivo*.

## Introduction

The altered metabolism of cancer cells characterized by high rates of glucose consumption and glycolysis was described by Otto Warburg eighty years ago [1]. The use of positron emmission tomography (PET) to detect tumors and/or metastatic lesions relies upon uptake of a radioactive glucose analog by tumors which also demonstrates the increased glucose utilization by the majority of tumors [2]. Tumor hypoxia (due to proliferation outpacing a blood supply) and subsequent activation of Hypoxia Inducible Factor (HIF) is certainly responsible for some of the increased glycolysis and glucose consumption observed in cancer cells since HIF activates transcription of a number of glycolytic genes as well as GLUT1 [3], [4], [5]. However, hypoxia cannot be completely responsible for the elevated glucose transport and increased glycolysis observed in tumor cells since these properties are induced by oncogene-mediated cell transformation *in vitro* under normoxic conditions [6], [7].

Glucose is transported into cells via two classes of hexose transporters: the GLUT family and the sodium-dependent glucose transporter (SGLT) family. The SGLT family of transporters transport sugars against the concentration gradient utilizing the sodium-electrochemical gradient and are predominantly expressed in the small intestine and the kidney [8], [9], although recent data suggests SGLT1 may be expressed in cancer cells that express the epidermal growth factor receptor (EGFR) [10]. The GLUT family includes fourteen hexose transporters (twelve in mouse) which are facilitative transporters that transport sugars along the concentration gradient [11], [12]. GLUT4 is the insulin responsive transporter and is expressed in adipose tissue and skeletal muscle [13]. The most widely expressed hexose transporter is GLUT1 which is thought to maintain basal glucose transport in most types of cells [11], [12]. GLUT1 also appears to be the predominant glucose transporter in many types of cancer cells [14], [15], [16], [17], [18], [19], including breast cancer, although expression of GLUT2, GLUT3, GLUT5, GLUT6 and GLUT12 have been detected in cancer cells using immunohistochemistry or RNA analysis [14], [16], [18], [20]. Additionally, the expression of GLUT1 often correlates with the ability to detect tumors by PET [21], [22], [23].

We previously described high levels of lactate (indicative of aerobic glycolysis), low concentrations of glucose (indicative of high rates of glucose consumption) and elevated expression of GLUT1 in mammary tumors from MMTV-c-ErbB2 mice and that these changes were further enhanced in tumors that arose in the bitransgenic MMTV-c-ErbB2, MMTV-myr-Akt1 mice [24]. We hypothesize that GLUT1 is a critical mediator of cell survival, proliferation, glucose uptake and aerobic glycolysis in breast cancer cells *in vitro* and in mammary tumors *in vivo*, and tested this hypothesis using ErbB2 or PyVMT overexpressing mouse mammary carcinoma cell lines and tumors.

## Materials and Methods

### Ethics Statement

All mice were maintained in the Center for Comparative Medicine at the University of Colorado Denver - Anshutz Medical Campus, an AALAC-approved facility. Additionally, the University of Colorado Denver Institutional Animal Care and Use Committee specifically approved this study and the protocols used during this study (Protocol #23103607(11)2E).

### Cell Culture

Mouse mammary tumor cell lines (78617, 78622, 78717, 85815 and 85819) generated from tumors arising in MMTV-c-ErbB2 mice were obtained from the laboratory of Ann Thor (University of Colorado School of Medicine) [25]. 293T and Bosc packaging cells were obtained from the Tissue Culture Core, University of Colorado Cancer Center. Met1 cells were provided by Haihua Gu (University of Colorado School of Medicine). Other cell lines were from laboratory stocks. All cell lines were cultured in DMEM containing 10% fetal bovine serum, 40 ng/ml insulin, 1× non-essential amino acids and 100 units/ml penicillin, 100 µg/ml streptomycin. Fetal calf serum was obtained from Hyclone (Logan, UT). All media components were from Invitrogen (Carlsbad, CA) or Sigma-Aldrich (St. Louis, MO).

### Expression Vectors and Virus Preparation

pLKO.1 lentiviral plasmids containing shRNA constructs targeting mouse GLUT1 or control shRNA were from Open Biosystems (Huntsville, AL). The human *GLUT1* gene was removed from pSPMM1 [26] (provided by Mike Muekler; Washington University, St. Louis, MO) as BamH1/BamH1 fragment and subcloned into the pQCXIP retroviral vector (Clonetech; Mountain View, CA). The *GFP-luciferase* gene was removed from pEGFP-LUC2 (provided by Chuan-Yuan Li; University of Colorado School of Medicine) as a Not1/HindIII fragment and subcloned into the pLNCX2 retroviral vector. The pMIG-PyVMT plasmid [27] was provided by Heide Ford, University of Colorado School of Medicine. Lentivirus was prepared by cotransfecting 10 cm plates of 293T cells with 10.7 µg pLKO.1 proviral plasmid, 8 µg pΔ8.9 helper plasmid and 5.3 µg pVSVG envelope plasmid using the calcium phosphate method. Retrovirus was prepared by cotransfecting 60 mm plates of Bosc packaging cells with 2 µg retroviral plasmid and 1 µg pCL-Eco plasmid. Virus containing supernatants were harvested after 48 hours, filtered through a 0.45 µm filter and target cells infected in the presence of 8 µg/ml polybrene. Drug-resistant cells were selected 48 hours after infection with either 2 µg/ml puromycin or 0.5 mg/ml G418. Adenovirus expressing GFP (Ad-GFP) or adenovirus expressing Cre recombinase (Ad-Cre) were provided by Jerome Schaack (University of Colorado School of Medicine). Adenovirus particle concentrations were determined spectrophotometrically, with one A_260_ unit considered equal to 10^12^ particles (or 10^10^ plaque forming units) as described previously [28].

### Generation of Mice with Floxed GLUT1 Allele

The murine GLUT1 (slc2a1) locus was isolated from a bacterial artificial chromosome (BAC) clone that was obtained from Clontech. The source of the DNA was a male CJ7/129SV. A 9.1 kb fragment from EcoR1 (8055) to HindIII (17172) that encompasses intron 2, exons 3–10 and intron 10 of the GLUT1 locus was subcloned. The 5′ loxP site was subcloned into an Nco1 site (11074) in intron 2. An frt-Neo-frt-loxP cassette was subcloned into an EcoR1 site (14615) located in intron 9. The final targeting vector was subcloned into the TK1-TK2 C vector containing 2 thymidine kinase cassettes in the tandem with the vector. The linearized vector was electroporated into isogenic W9.5 embryonic stem cells at the Embryonic Stem Cell Laboratory in the Section of Comparative Medicine, Yale University School of Medicine. 276 Neo resistant clones were screened and one recombinant ES cell clone was obtained and homologous integration was confirmed by Southern blot and PCR (Figure S2). This ES cell clone was expanded and injected into the blastocysts of C57BL6 mice. Chimeric mice were obtained and germline transmission was confirmed by Southern blotting. Heterozygous offspring harboring the modified recombinant (loxP-frt-Neo-frt-loxP) GLUT1 allele were mated with transgenic mice with germline expression of the FLPe recombinase and offspring were screened for deletion of the frt flanked Neomycin cassette, but with retention of the 3′ loxP site. Heterozygous offspring for the floxed GLUT1 allele were then bred to homozygosity. Maps of the targeted allele and the genotyping scheme are summarized in l Figures S2, S3.

### Generation of G1fP and G1fPt cells

Late pregnant or lactating mice with exons 3–8 of the *GLUT1* gene“floxed” were euthanized and the #4 mammary glands surgically excised, minced, digested with 1 mg/ml collagenase and cultured as described [29] before being infected with polyomavirus middle T antigen (PyVMT) expressing retrovirus (pMIG-PyVMT [27]) to immortalize and transform the cell line. The resulting cell lines were passaged over twenty times to establish stably proliferating cell lines: “GLUT1 ^fl/fl^ PyVMT mammary cells” or “G1fP” cells. G1fP cells were infected with Ad-GFP or Ad-Cre at a multiplicity of infection (MOI) of 100 prior to carrying out biochemical studies. G1fP cells which had not been exposed to Cre recombinase were infected with a luciferase expressing retrovirus and injected into the mammary fat pad of athymic nude mice, as described below, and the resulting tumors were used to generate tumor cell lines in the same manner described above, one of which is called “G1fPt” cells (the added “t” is to denote that the cells were reisolated from a tumor).

### cDNA Synthesis and qPCR Analysis

RNA was isolated from homogenized tumor samples using Trizol reagent (Invitrogen; Carlsbad, CA) and from cultured cells using RNeasy Plus Mini kit (Qiagen; Germantown, PA) following manufacturers' instructions. 2 µg total RNA was subjected to single-strand cDNA synthesis using 2 µM random hexamers, 20 µg/ml oligo-dT and MuMLV reverse transcriptase. qPCR was performed with a dilution of cDNA equivalent of 50 ng RNA and the following mouse primer/probe sets (Applied Biosystems; Foster City, CA): GLUT1–GLUT10, GLUT12, GLUT13, SGLT1, RPL32 and β-actin. The best fit linear equation generated by the amplicon standard curve (1.204×10^7^ to 7.71×10^2^ copies/µl) was used to determine the number of copies of GLUT1, GLUT6, GLUT8 or GLUT9 in the cDNA generated from 50 ng RNA. Relative transporter expression was normalized to the expression of β-actin in preliminary experiments or ribosomal protein L32 (RPL32) in later experiments (and RPL32 expression was unaltered between the groups being compared). % of RPL32 gene expression was calculated using the equation: (2^ΔCt^)(100) where ΔCt = [Ct RPL32−Ct GLUT]. % β-actin expression was calculated the similarly.

### Immunoblot analysis

Protein was extracted from minced tumor tissue homogenized using a polytron or from plates of cultured cells and immunoblot analysis was performed as previously described [24] using the following antibodies: anti-GLUT1 (Millipore; Billerica, MA or AbCam; Cambridge, MA); anti-cytokeratin 18, anti-GFP and anti-β-actin (Santa Cruz Biotechnology; Santa Cruz, CA).

### Glucose Consumption, Lactate Secretion and ATP Assays

Cells were cultured in 24 well plates (200,000 cells/well) for 18 hours, and the glucose and lactate in the conditioned media was quantified using the Glucose Assay Kit (Sigma-Aldrich; St. Louis, MO) and the Lactate Assay Kit (BioVision; Mountain View, CA) following manufacturers' instructions. The difference in the amount of glucose in each sample compared to the amount of glucose in media incubated without cells reflected glucose consumption. Quantities of glucose consumed and lactate secreted were normalized to the DNA content of each well and quadruplicate samples were analyzed. The ATP content of cells was determined using the ATPlite Luminescence Assay (Perkin Elmer; Walthan, MA) following manufacturer's instructions.

### Proliferation and DNA Quantitation Assay

10,000 cells were added per well of 24 well plates in quadruplicate and were cultured for 0–4 days. DNA concentrations of the monolayers collected on successive days were determined using the Hoechst 33258 assay [30] as a means to determine relative proliferation. The DNA concentration was also used to normalize glucose consumption, lactate secretion or ATP concentration data.

### ^3^H -2-deoxyglucose Transport Assay

Cells in 24 well plates (200,000 cells/well) were seeded for 18 hours, washed with PBS, incubated for 1 hour in glucose-free DMEM, and then pulsed with 2 µCi of ^3^H-2-deoxyglucose (∼60 pmol) (PerkinElmer; Waltham, MA) for 15 minutes. The monolayers were washed three times with ice cold PBS, lysed with 0.5 ml 1 M NaOH, neutralized with 0.5 ml 1 M HCl, and quantitated by liquid scintillation counting. DNA concentrations of non-radioactive control wells were used to normalize radioactivity incorporation and data is presented as CPM per µg DNA.

### Lipid Synthesis Assay

500,000 cells in complete media with 1 mg/ml glucose containing 500 nCi ^14^C-U-glucose (Perkin Elmer, Walthan, MA) were incubated overnight. Media was removed and monolayers were lysed with 100 µl 0.5% Triton-X-100 and transferred to a microfuge tube. Methanol∶chloroform extraction was performed by sequentially adding 250 µl methanol, 250 µl chloroform, 250 µl chloroform and 250 µl 0.9% NaCl with vortexing between each step. The lower lipid containing chloroform phase was transferred to a scintillation vial and the sample was re-extracted with an additional 500 µl chloroform which was transferred to the same vial. Chloroform was evaporated under a stream of nitrogen, the lipid dissolved in 100 µl ethanol and 5 ml scintillation cocktail and quantitated by liquid scintillation counting. DNA concentrations of non-radioactive control plates were used to normalize radioactivity incorporation.

### Growth in Soft Agar

30,000 cells were resuspended in 10 ml of 0.4% agar containing 1× DMEM/F12 and 10% FBS, and 3 ml were added to three different agar coated wells of six well plates. Media was changed twice weekly, and after three weeks the plates were photographed with a BioRad Gel Doc imaging system (Hercules, CA) and colonies quantitated using the colony counting tool (Quantity One Software). One hour prior to harvest the media was supplemented with 3 µg/ml BrdU. The plates were washed with PBS and plugs of soft agar were removed, wrapped in lens paper, fixed for 1 hour in 10% neutral buffered formalin, processed in a tissue processor and embedded in paraffin.

### Tumor cell transplantation

Tumor cell lines expressing GFP-luciferase or luciferase were resuspended in 50% matrigel (BD biosciences; Bedford, MA), 50% PBS to a concentration of 0.4 or 0.5 million cells per 20 µl. 20 µl cells were back-loaded to insulin syringes and injected into surgically exposed #4 mammary glands of athymic nude mice (Jackson Laboratories; Bar Harbor, ME) anesthetized with ∼2 mg Avertin (Sigma-Aldrich; St. Louis, MO). Tumor growth was monitored by bioluminescence by intraperitoneal injection of 200 µl sterile 15 mg/ml D-luciferin (Gold Biotechnology; St. Louis, MO) followed by anesthetization with 2% isoflourane and imaging with an IVIS 200 Bioimager (Small Animal Imaging Core, University of Colorado Cancer Center) seven minutes after D-luciferin administration. Bioluminescence data was evaluated using Living Image 2.60.1 software. Two hours prior to sacrifice and tumor harvest, mice were injected with 500 µl 3 mg/ml BrdU (GE Healthcare; Pittsburgh, PA).

### Histological Analyses

Dissected tumors were fixed in 10% neutral buffer formalin and processed by the Pathology Core Facility of the University of Colorado Cancer Center. Antigen retrieval was performed by heating slides in 10 mM citrate pH 6.0 for 20 minutes in a microwave. Slides were blocked with 10% normal goat serum and incubated overnight at 4°C with GLUT1 antibody (Global Peptide; Fort Collins, CO [24]) diluted to 0.1 µg/ml or rabbit anti-Ki67 antibody (Abcam; Cambridge, MA) diluted 1∶75. Biotinylated goat anti-rabbit secondary antibody and tertiary Vector ABC or ABC elite (Vector Laboratories, Burlingame, CA) was followed by DAB color development and hematoxylin counterstaining. Detection of apoptotic cells was performed similarly using SignalStain cleaved caspase-3 IHC detection kit (Cell Signaling Technologies, Beverly, MA) with pre-diluted rabbit anti-cleaved capsase-3 antibody. Proliferating cells that had incorporated BrdU were identified using the anti-BrdU kit from BD Biosciences following manufacturer's instructions. Light photomicrographs were captured using an Olympus BX40 microscope equipped with a Spot Insight QE camera and Spot Advanced software. Proliferation was quantitated by determining the total number of nuclei and BrdU-positive or Ki67-positive nuclei in three-four high power fields in four tumor sections of each group to determine the percent of positively staining nuclei. Twelve BrdU stained soft agar colonies per group were analyzed similarly, but only the outer two cell layers of each colony was included in the quantification. Five high power photomicrographs of “hot spots” of cleaved caspase 3 staining were converted to binary pictures using ImageJ software to quantitate the number of pixels representing cleaved caspase 3 positive staining in four tumors from each group.

### Statistical Analyses

All data are presented as the mean +/− standard deviation and * is used to denote statistical significance (p<0.05) between groups as determined by two tailed T test assuming unequal variance when comparing two groups, unless otherwise indicated. Significant differences (p<0.05) between multiple groups were determined by ANOVA and Bonferroni post-hoc tests (multiple testing-corrected).

## Results

### GLUT1 is the most abundantly expressed hexose transporter in MMTV-c-ErbB2 mammary tumors and numerous mouse mammary carcinoma cell lines

**Figure 1:**
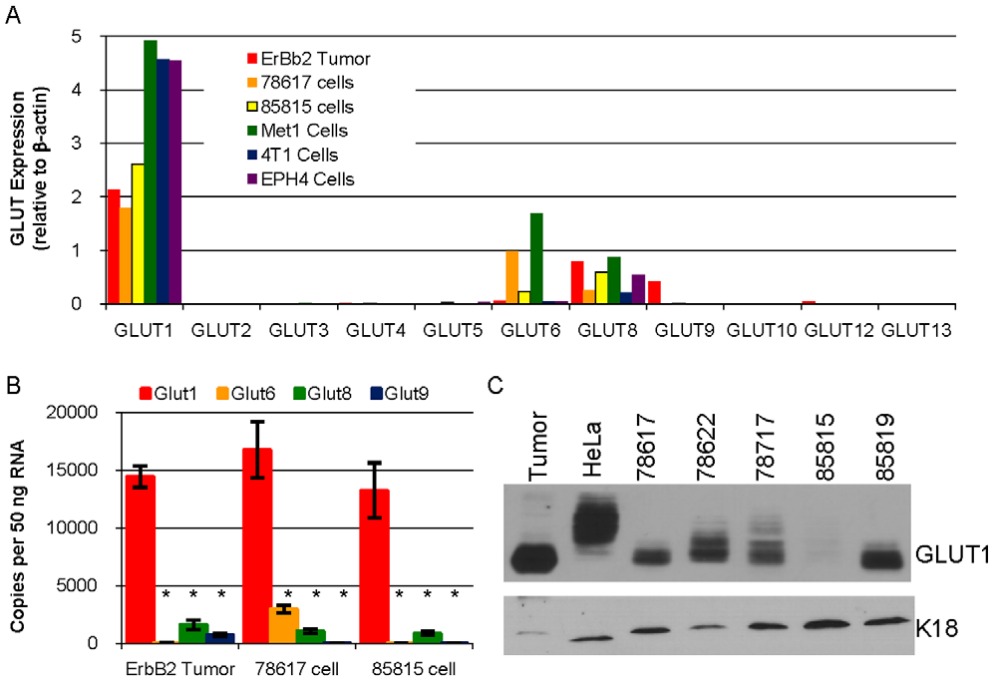
GLUT1 is the most abundantly expressed GLUT family member in MMTV-c-ErbB2 tumors and a number of mouse mammary carcinoma cell lines. **A.** qPCR analysis to determine the expression of GLUT1–GLUT6, GLUT8–GLUT10, GLUT12–GLUT13 (eleven of the twelve mouse GLUT family transporters) relative to β-actin expression was performed with the cDNA equivalent of 50 ng RNA in six samples: mammary tumor from a MMTV-c-ErbB2 mouse (ErbB2 tumor), two different cell lines derived from these tumors (78617 and 85815), a cell line derived from a MMTV-PyVMT mouse mammary tumor (Met1), a cell line derived from a BALB/c mouse mammary tumor (4T1) and immortalized mouse mammary epithelial cells (EPH4). **B.** Quantitation of the number of copies of GLUT1, GLUT6, GLUT8 and GLUT9 RNA in the cDNA derived from 50 ng RNA from triplicate samples of ErbB2 Tumors, 78617 cells and 85815 cells. * indicates p<0.05 for GLUT1 expression versus the other transporters as determined by Bonferroni post-hoc tests. **C.** Immunoblot analysis of GLUT1 and keratin 18 (K18) in lysates of an MMTV-c-ErbB2 tumor (tumor), HeLa cells, and five different MMTV-c-ErbB2 tumor cell lines (78617, 78622, 78717, 85815 and 85819).

**Figure 2:**
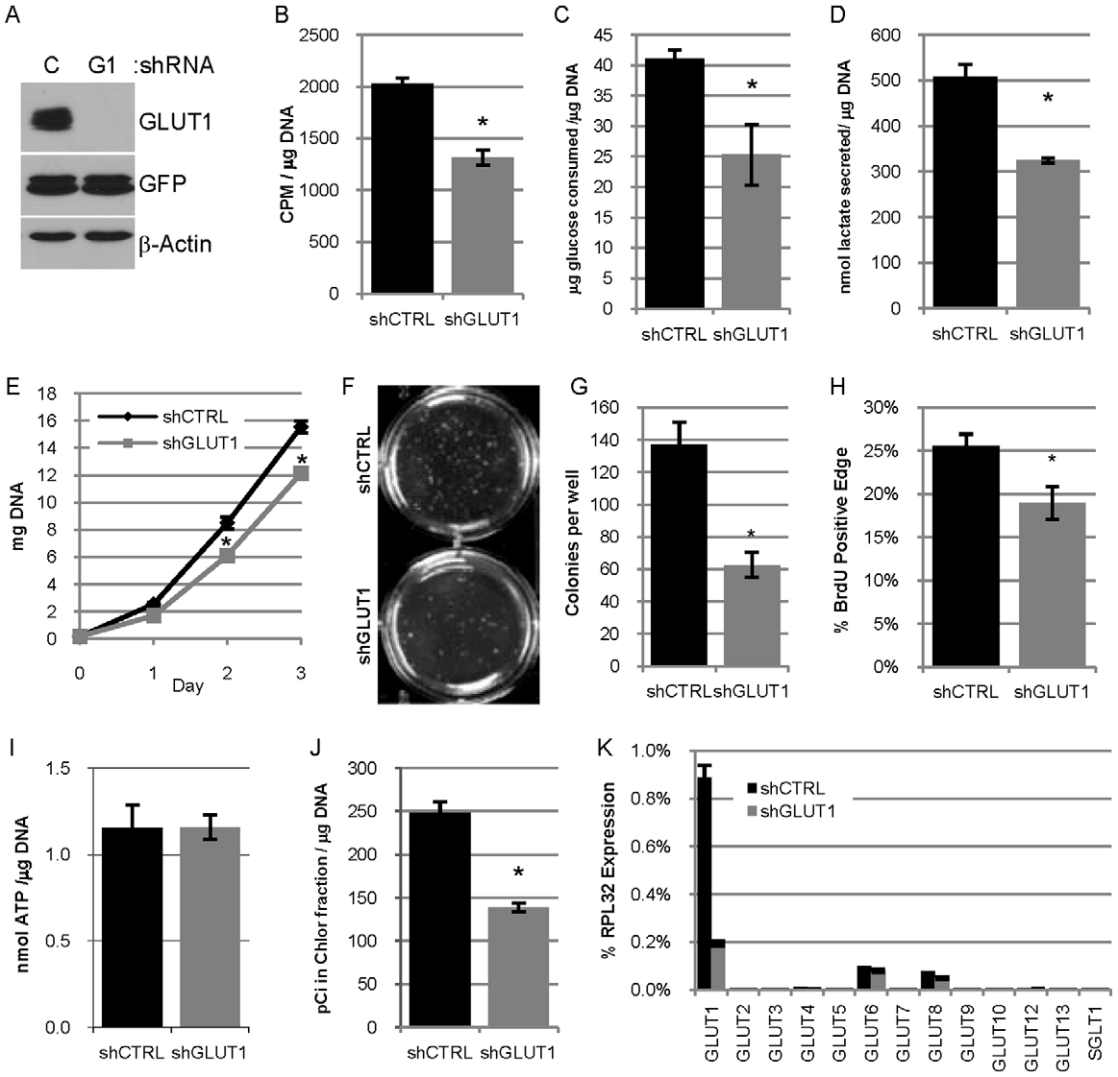
Reduced expression of GLUT1 in 78617GL cells decreases glucose usage, lipid synthesis and proliferation in vitro. **A.** Immunoblot analysis evaluating the expression of GLUT1, GFP-Luciferase transgene (GFP) and β-actin in lysates from 78617GL cells expressing control shRNA (C) or GLUT1 shRNA (G1). **B.** Uptake of ^3^H-2-deoxyglucose by 78617GL cells expressing control shRNA (shCTRL) or GLUT1 shRNA (shGLUT1) in 15 minutes presented as CPM per µg DNA. **C–D.** Glucose consumption (**C**) and lactate secretion (**D**). Glucose and lactate concentrations are normalized to the DNA content of the cultures. **E.** Proliferation is estimated by deteriming the DNA content of cultures at days 0, 1, 2 and 3 post-seeding. **F–G.** 78617GL cells were grown in soft agar for 3 weeks and colonies are pictured in **F** and the number of colonies per well is quantified in **G**. **H.** Quantification of BrdU positive cells in the outer edge of 12 colonies of each group. **I.** The concentration of ATP in the two groups of cells (lacking luciferase expression) was determined and normalized to the DNA content of parallel monolayers. **J.** Lipid synthesis was measured by determining the amount of ^14^C in the non-aqueous chloroform fraction of methanol chloroform extracted cell lysates after 24 hour incubation with ^14^C-glucose and is normalized to the DNA content of parallel samples. **K.** qPCR analysis evaluating the expression of the 12 mouse GLUT transporters and SGLT1 in 78617GL cells expressing control shRNA or GLUT1 shRNA normalized to RPL32 expression.

A qPCR screen evaluating the expression of eleven of the twelve mouse GLUT family members was performed with the following samples: tumors derived from MMTV-c-ErbB2 mice [24], two mammary tumor cell lines derived from such tumors (78617 and 85815 [25]), a mammary carcinoma cell line derived from MMTV-PyVMT mice (Met1 [31]), a BALB/c mouse mammary carcinoma cell line (4T1 [32]), and immortalized mouse mammary epithelial cell line (EPH4). This initial screen demonstrated that GLUT1 was the most abundantly expressed GLUT family member in ErbB2 overexpressing mouse mammary tumors and all five cell lines examined (Figure 1A). Other GLUT family members which were expressed included GLUT6, GLUT8 and GLUT9 (Figure 1A, Figure 2K). We further analyzed the expression of GLUT1, GLUT6, GLUT8 and GLUT9 in multiple samples of the ErbB2-overexpressing tumors and cell lines by performing qPCR against a standard curve for each transporter to determine the number of copies of each transporter, independent of any reference gene (Figure 1B). This analysis demonstrated that GLUT1 was the most highly expressed transporter in all samples and that all samples also expressed GLUT8, 78617 cells expressed GLUT6 and the ErbB2 tumors expressed GLUT9 (Figure 1B). These samples all lack expression of GLUT7 (data not shown, Figure 2K), the one GLUT family member not examined in our initial screen (Figure 1A).

Immunoblot analysis utilizing lysates from a primary MMTV-c-ErbB2 mouse mammary tumor and five cell lines derived from tumors taken from these mice (78617, 78622, 78717, 85815 and 85819) using HeLa cells as a control, revealed that all express GLUT1 protein (Figure 1C). Numerous immunoreactive bands are present in GLUT1 immunoblot analysis due to glycosylation of GLUT1 [33] and later experiments show all bands are reduced by RNAi or Cre recombinase and conversely all bands are present when GLUT1 is overexpressed. 85815 cells express very low levels of the GLUT1 protein demonstrating that GLUT1 protein levels do not necessarily correlate with the amount of RNA detected by qPCR (Figure 1A–B). Expression of keratin 18 (K18) demonstrates that the cell lines are of epithelial origin and all lanes are loaded similarly (Figure 1B).

### Reduced expression of GLUT1 in 78617GL cells decreases glucose usage, lipid synthesis and proliferation in vitro

We had previously noted increased lactate concentration, decreased glucose concentration and increased GLUT1 expression in tumors from MMTV-c-ErbB2 mice compared to mammary epithelial cells [24] and wanted to test whether manipulation of GLUT1 expression levels in ErbB2-overexpressing mammary carcinoma cells could modulate aspects of glucose usage, bioenergetics and biosynthesis *in vitro*. The 78617 cell line was transduced with a retrovirus encoding GFP-luciferase to create “78617GL” cells. These cells were transduced with lentiviruses expressing shRNA molecules targeting GLUT1 or a non-silencing control shRNA to generate pools of cells stably expressing each shRNA. Immunoblot analysis revealed that the GLUT1 shRNA effectively reduced GLUT1 protein expression as compared to cells expressing the control shRNA (Figure 2A). 78617GL cells with undetectable amounts of GLUT1 protein were viable and survived in culture for numerous passages and demonstrated no apparent increase in caspase 3 activation (data not shown). There was also no difference in the expression of numerous Bcl2 family members, including PUMA (data not shown), which has been shown to be increased under conditions of low glucose to mediate p53-dependent cell death [34].

We sought to determine whether these cells with reduced GLUT1 protein have altered glucose usage. There is a 35% decrease in the uptake of ^3^H-2-deoxyglucose (^3^H -2-DOG) in 78617GL cells expressing GLUT1 shRNA compared to the cells expressing control shRNA (Figure 2B). Similarly, 78617GL cells expressing control shRNA consume more glucose and secrete more lactate than 78617GL cells expressing GLUT1 shRNA (Figure 2C–D). These data demonstrate that reduction of GLUT1 expression leads to a reduction of glucose transport, glucose consumption and lactate secretion suggestive of reduced glycolysis and glucose metabolism.

We wanted to determine if decreasing GLUT1 expression would reduce proliferation since cell division requires energy (ATP) and carbon for macromolecule biosynthesis, both of which can be supplied by glucose. 78617GL cells expressing GLUT1 shRNA had a reduced rate of proliferation as compared to the cells expressing the control shRNA, which was apparent on days 1, 2, and 3 (Figure 2E). Similarly, 78617 cells expressing GLUT1 shRNA formed fewer colonies when grown in soft agar (Figure 2F–G, Figure S1). Colonies from cells expressing control shRNA and GLUT1 shRNA had similar patterns of cleaved caspase 3 staining: larger colonies tended to have staining at the core of the colony while medium and small colonies tended to have very little cleaved caspase 3 staining (Figure S1), though the colonies from shGLUT1 cells did appear to have more frequent central apoptosis. Nuclear BrdU incorporation tended to occur at the perimeter of the colonies (Figure S1) and 78617GL cells expressing control shRNA had a higher percentage of BrdU positive nuclei at their edge than colonies derived from cells expressing GLUT1 shRNA (Figure 2H and Figure S1) suggesting cells with reduced GLUT1 have reduced proliferation in soft agar. 78617 cells expressing GLUT1 shRNA or control shRNA (grown on plastic) had identical concentrations of ATP (Figure 2I) suggesting that impaired glucose usage and GLUT1 expression does not deplete ATP under the conditions of this study. However, 78617GL cells expressing GLUT1 shRNA cultured with radiolabeled glucose convert less of the aqueous label to the non-aqueous lipid containing fraction than cells expressing control shRNA (Figure 2J), suggesting reduced lipid synthesis in cells expressing GLUT1 shRNA.

The shRNA mediated reduction of GLUT1 appears to eliminate detectable GLUT1 protein by immunoblot analysis (Figure 2A), but 78617GL cells expressing GLUT1 shRNA still transport and consume glucose (Figure 2B–C), albeit at a lower rate than the control cells. qPCR analysis reveals that GLUT1 is still the most highly expressed GLUT family member in cells expressing GLUT1 shRNA and that no other GLUT family member or SGLT1 is being upregulated (at the RNA level) in cells lacking detectable GLUT1 protein (Figure 2K). Thus, glucose consumption by cells expressing GLUT1 shRNA may still occur via remaining GLUT1 (or other transporters) or by non-specific means such as pinocytosis.

### Reduced expression of GLUT1 decreases tumor growth in nude mice

**Figure 3:**
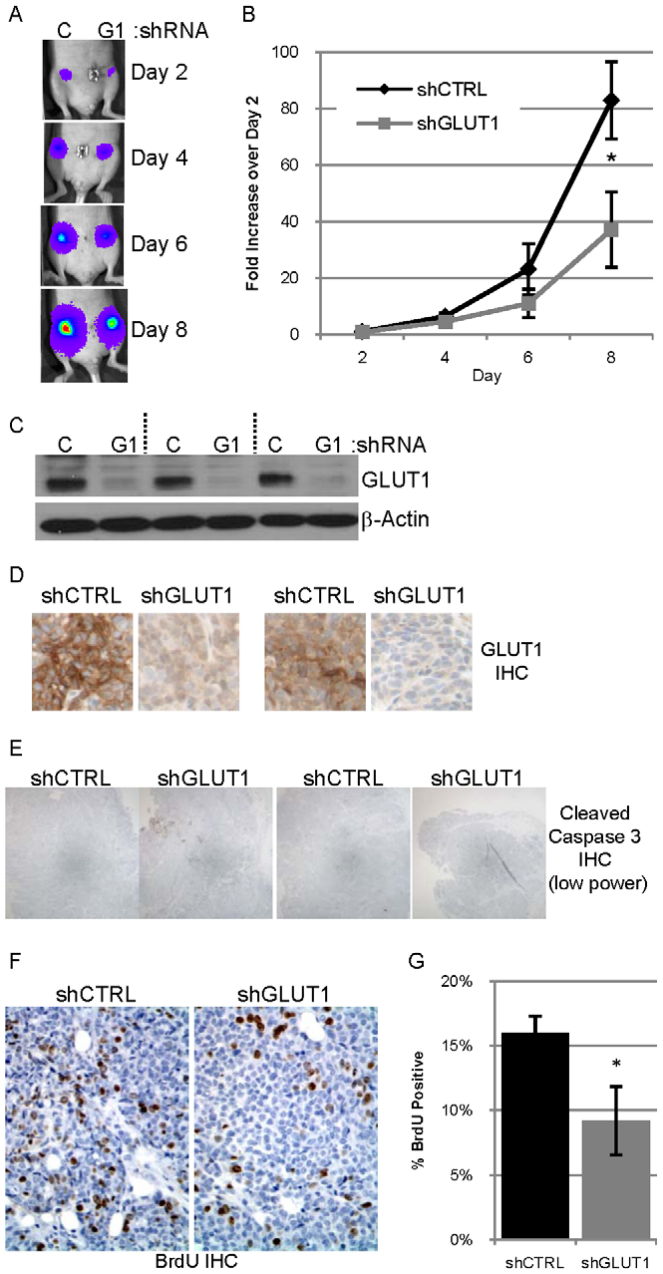
Reduced expression of GLUT1 in 78617GL cells decreases tumor growth. **A.** 0.5 million 78617GL cells expressing control shRNA (C) or GLUT1 shRNA (G1) were injected into contralateral #4 mammary fat pads of athymic nude mice. Bioluminescence from the labeled tumor cells was detected on days 2, 4, 6 and 8 after implantation. The abdominal region heat map depicting luciferase activity of a representative mouse is pictured. **B.** The average bioluminescence on days 2, 4, 6 and 8 normalized to the day 2 bioluminescence +/− SEM for five mice is presented with the black diamonds representing shCTRL tumors and grey squares representing shGLUT1 tumors. **C.** Expression of GLUT1 and β-actin in lysates of three tumor pairs evaluated by immunoblot analysis. **D.** GLUT1 expression evaluated by IHC (with hematoxylin counterstain) in two tumor pairs. **E.** Representative low power photomicrographs of two pairs of tumor sections immunostained for cleaved caspase 3. **F–G.** Representative high power photomicrographs of a tumor pair immunostained for BrdU with hematoxylin counterstain (**F**) which is quantified (**G**).

To address the role of GLUT1 in tumor growth *in vivo*, 78617GL cells expressing control shRNA or GLUT1 shRNA were transplanted into contralateral #4 mammary glands of five athymic nude mice and tumor growth was monitored by bioluminescence on days 2, 4, 6, and 8 post tumor cell transplant (Figure 3A) which is quantitated and averaged for all five mice (Figure 3B). In each mouse, the tumor derived from cells expressing GLUT1 shRNA was smaller than the contralateral tumor derived from cells expressing control shRNA, and this difference was most evident on days 6 and/or 8 (Figure 3A and data not shown), and this is also true of the average across all five mice (Figure 3B). Four of the five tumors derived from cells expressing control shRNA outweighed the contralateral tumor expressing GLUT1 shRNA (with the fifth tumor pair weighing the same) (data not shown), which corroborates the bioluminescence data by suggesting decreased growth of cells expressing GLUT1 shRNA *in vivo*. A separate, similar experiment utilizing five mice corroborated these results: 78617GL cells expressing GLUT1 shRNA are smaller than control tumors in their initial ten days of growth (data not shown).

Immunoblot analysis of tumor lysates and immunohistochemical staining of tumor sections reveals that tumors derived from 78617GL cells expressing GLUT1 shRNA maintain low levels of GLUT1 protein (Figure 3C–D); however this GLUT1 protein may reflect either endothelial cells or infiltrating immune cells present in the tumors analyzed. qPCR analysis revealed that there was no change in the levels of other GLUT family members between the two tumor types suggesting that the low level of GLUT1 was not compensated for by an increase in the expression of these other GLUTs (data not shown), similar to what was observed with these cell lines *in vitro* prior to transplantation into mice (Figure 2K).

Surprisingly, tumors derived from cells expressing control shRNA and GLUT1 shRNA both revealed very little staining for cleaved caspase 3 (Figure 3E). Quantification of cleaved caspase 3 staining revealed no difference in apoptosis between the two tumor types (data not shown). Quantification of the percentage of BrdU positive nuclei in 3 high power images from four tumors of each type revealed that tumors derived from cells expressing GLUT1 shRNA had a lower rate of proliferation than the control tumors (Figure 3F–G). This suggests that the differences in tumor size more likely reflect differences in proliferation than differences in apoptosis. Since these tumors were harvested at an early stage when they weighed ∼200 mg, there was no central necrosis observed.

### Eliminating GLUT1 expression in G1fP cells decreases glucose usage, lipid synthesis and proliferation in vitro

**Figure 4:**
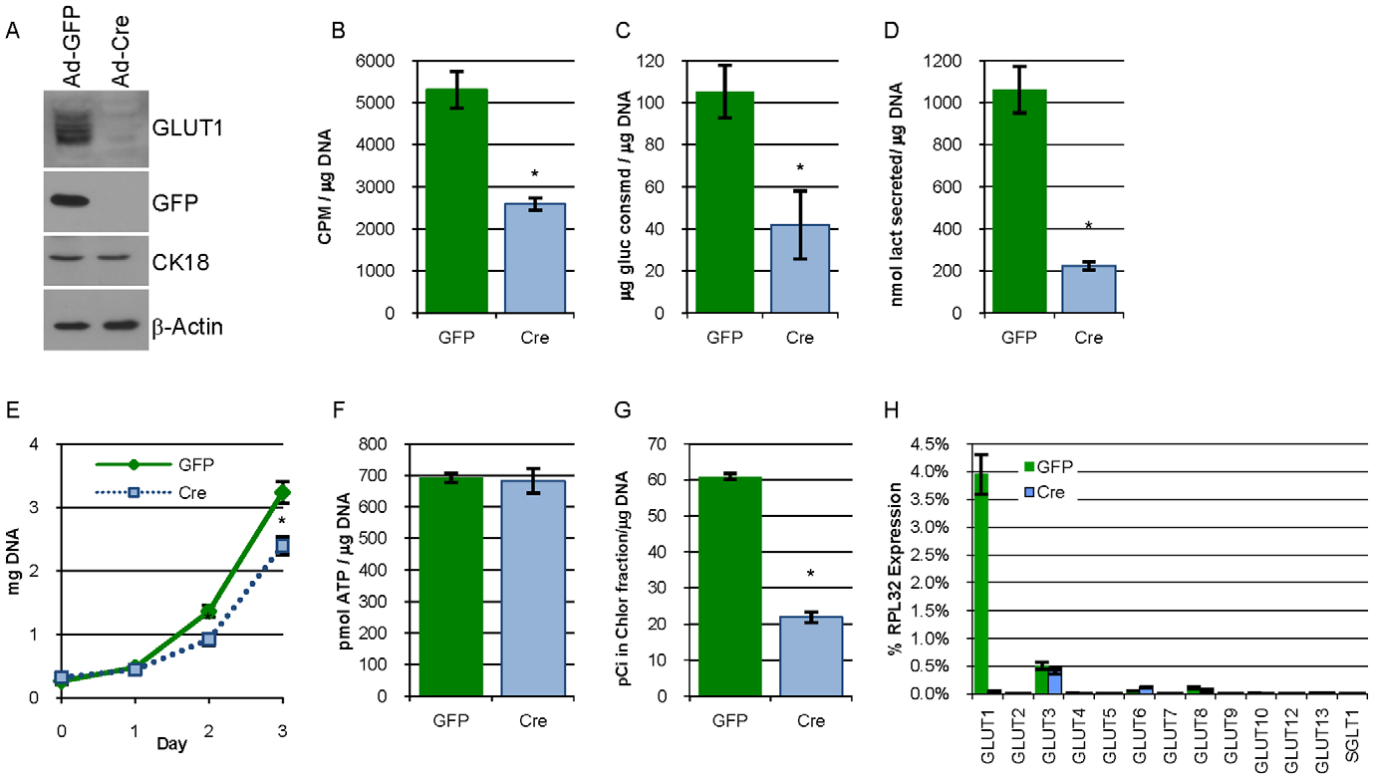
Eliminating expression of GLUT1 in G1fP cells decreases glucose usage, lipid synthesis and proliferation in vitro. **A.** Immunoblot analysis evaluating the expression of GLUT1, GFP, CK18, and β-actin in lysates from GLUT1 ^fl/fl^ mammary cells transformed with PyVMT (G1fP cells) 72 hours after being infected with adenovirus expressing GFP (Ad-GFP) or Cre recombinase (Ad-Cre) at an MOI of 100. **B–D.** Uptake of ^3^H-2-deoxyglucose **(B)**, glucose consumption (**C**), and lactate secretion (**D**) by G1fP cells previously infected with Ad-GFP or Ad-Cre as described in figure 2. **E.** Proliferation is estimated by determining the DNA content of cultures at days 0, 1, 2 and 3 post-seeding. **F.** The concentration of ATP in the two groups of cells was determined and normalized to the DNA content of parallel monolayers. **G.** Lipid synthesis in G1fP cells was measured as described in figure 2. **H.** qPCR analysis evaluating the expression of the 12 mouse GLUT transporters and SGLT1 normalized to RPL32 expression in G1fP cells that had been infected two weeks prior with Ad-GFP or Ad-Cre.

Use of RNAi reduced, but did not eliminate GLUT1 expression in 78617GL cells, so we generated a system in which we could eliminate GLUT1 expression. Harvested mammary epithelial cells from GLUT1^fl/fl^ mice (Figures S2, S3) were transformed in culture with the polyomavirus middle T antigen, an oncogene that shares many similarities to ErbB2 and is frequently used in mouse models of breast cancer [35], to establish a stably proliferating cell line named “G1fP” (for Glut1 floxed PyVMT. G1fP cells were infected at an MOI of 100 with adenovirus expressing GFP or Cre recombinase which resulted in the expression of GFP and the elimination of detectable GLUT1, respectively (Figure 4A). Expression of CK18 in these cells demonstrates their epithelial origin and equal loading (Figure 4A). G1fP cells exposed to Cre recombinase had a 60% reduction in ^3^H -2-DOG transport, a 60% reduction in glucose consumption and an approximate 80% reduction in lactate secretion as compared to cells exposed to GFP adenovirus (Figure 4B–D). G1fP cells lacking GLUT1 also had a reduced rate of proliferation (Figure 4E). While there was no difference in the ATP content of G1fP cells expressing GLUT1 or lacking GLUT1 expression (Figure 4F), the cells lacking GLUT1 expression had a greater than 60% decrease in the flux of glucose into lipid (Figure 4G). The expression of the GLUT family of transporters and SGLT1 evaluated by qPCR revealed that this cell line preferentially expressed GLUT1 with low level expression of GLUT3, GLUT6, and GLUT8, but removal of GLUT1 by Cre recombinase did not increase the expression of any of the other remaining hexose transporters (Figure 4H).

### Eliminating expression of GLUT1 decreases tumor growth in nude mice

**Figure 5:**
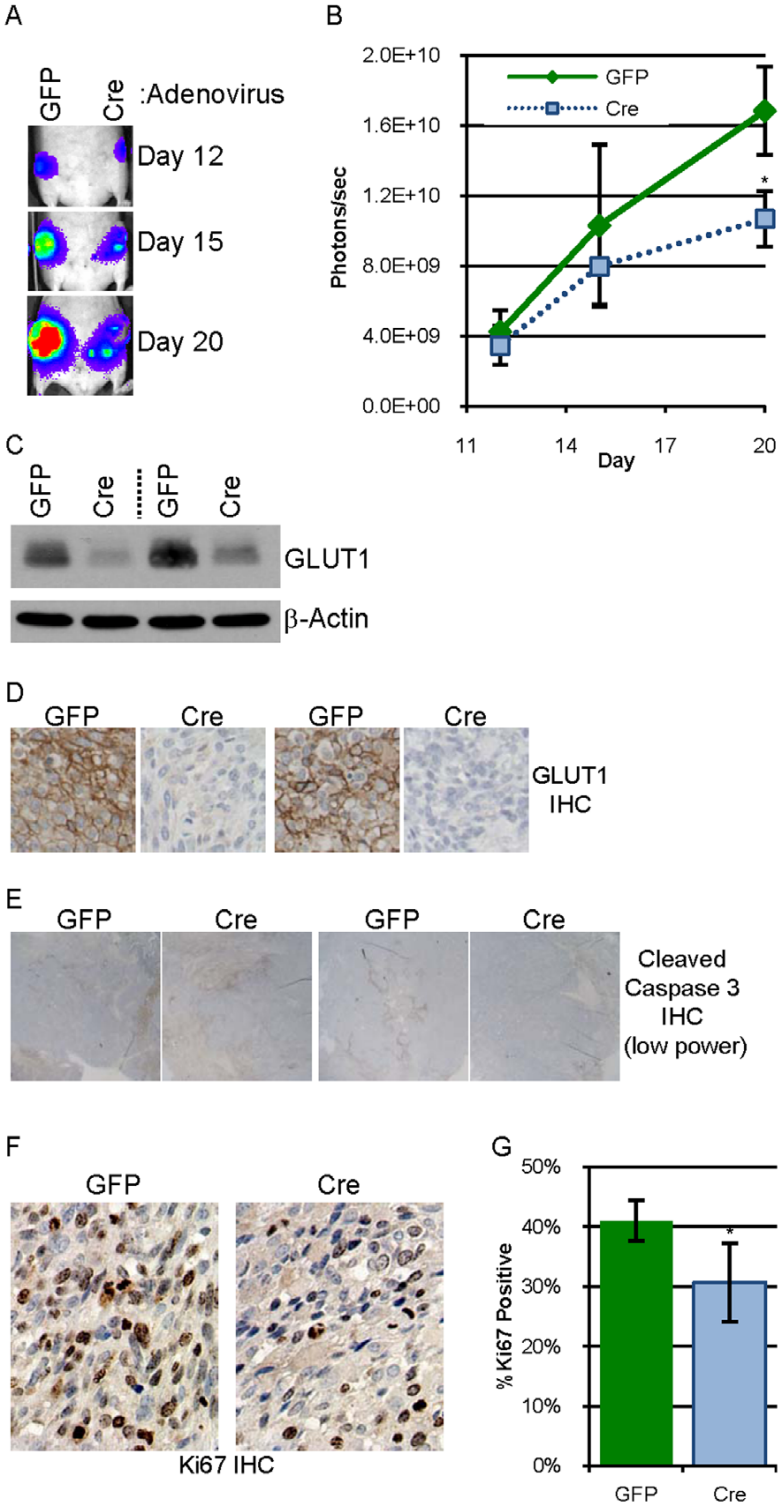
Elimination of GLUT1 expression in G1fPt cells decreases tumor growth. **A.** 0.5 million G1fPt cells infected two weeks prior with adenovirus expressing GFP or Cre recombinase were injected into contralateral #4 mammary fat pads of athymic nude mice. Bioluminescence from the labeled tumor cells was detected on days 12, 15 and 20 after implantation. The abdominal region heat map depicting luciferase activity of a representative mouse is pictured. **B.** The average bioluminescence on days 12, 15 and 20 +/− SEM for eight mice is presented with green diamonds representing “GFP” tumors and light blue squares representing “Cre” tumors. **C.** Expression of GLUT1 and β-actin in lysates of two tumor pairs evaluated by immunoblot analysis. **D.** GLUT1 expression evaluated by IHC (with hematoxylin counterstain) in two tumor pairs. **E.** Representative low power photomicrographs of two pairs of tumor sections immunostained for cleaved caspase 3. **F–G.** Representative high power photomicrographs of a tumor pair immunostained for Ki67 with hematoxylin counterstain (**F**) which is quantified (**G**).

To test whether G1fP cells, which were transformed in culture, are capable of tumor formation, G1fP cells expressing luciferase exposed to Ad-GFP or Ad-Cre were injected into contralateral #4 mammary glands of three athymic nude mice. In all three mice, the cells which had been exposed to GFP formed tumors with latencies ranging from 55–120 days while the mammary glands injected with cells exposed to Cre recombinase had no palpable tumor at the time of sacrifice or by later histologic examination (data not shown). A tumor cell line, “G1fPt” (for G1fP tumor), was generated from the first tumor arising from injected Ad-GFP infected G1fP cells. G1fPt cells were expanded in culture and then exposed to Ad-GFP or Ad-Cre and lysates evaluated by immunoblot analysis as described to confirm GLUT1 expression and elimination (data not shown). G1fPt cells exposed to Ad-GFP or Ad-Cre were injected into contralateral mammary glands of eight athymic nude mice and tumor development was monitored by bioluminescence on days 12, 15 and 20 post-injection (Figure 5A). The average bioluminescence from the two tumor types was similar on day 12, but by day 20, the tumors from the cells exposed to Cre were more than 35% smaller than the tumors with GLUT1 intact (Figure 5B). Similarly, in six of the eight harvested tumor pairs, the tumor from cells exposed to GFP weighed more than the tumor from cells exposed to Cre; and only one Cre tumor weighed more than the contralateral GFP tumor (data not shown). Examination of GLUT1 expression in the tumors by immunoblot analysis and IHC demonstrates that the tumors from Ad-Cre infected cells maintain reduced GLUT1 expression as compared to the control GFP tumors (Figure 5C–D). Both tumor types had similar, low levels of cleaved caspase 3 staining (Figure 5E). Tumor sections were stained for Ki67 (Figure 5F), a proliferation marker, and tumors from cells exposed to Cre recombinase had a 25% lower proliferative index than the tumors exposed to GFP (Figure 5G). These results corroborate the data from 78617GL cells: cells with reduced GLUT1 have reduced proliferation and form tumors more poorly than the control cells.

### Overexpression of GLUT1 in 85815GL cells increases glucose transport without increasing proliferation

85815 cells contain low levels of GLUT1 protein compared to the other mouse mammary tumor cell lines examined (Figure 1B) and reduction of GLUT1 in 85815 cells does not reduce proliferation of these cells *in vitro* or tumor growth initiated by these cells *in vivo* (data not shown). To ask whether increasing the level of GLUT1 in these cells would enhance proliferation and tumor growth *in vivo*, GLUT1 was overexpressed in 85815 cells expressing GFP-luciferase (85815GL cells).

**Figure 6:**
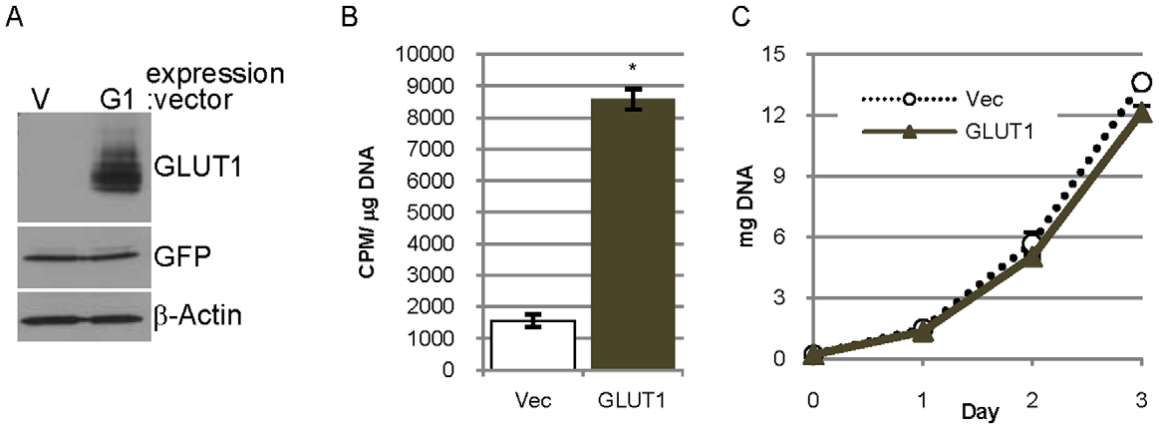
Overexpression of GLUT1 in 85815GL cells increases glucose transport without increasing proliferation. **A.** Expression of GLUT1, GFP-luciferase and β-actin in lysates of 85815GL cells expressing empty vector (V) or overexpressing GLUT1 (G1). **B.** Uptake of ^3^H-2-deoxyglucose by cells expressing empty vector (Vec) or GLUT1 (GLUT1) in 15 minutes presented as CPM per µg DNA. **C.** Proliferation is estimated by determining the DNA content of cultures expressing empty vector or GLUT1 at days 0, 1, 2 and 3.

85815GL cells were infected with a retroviral vector encoding human GLUT1 cDNA followed by selection in puromycin-containing media. Immunoblot analysis of lysates from these cells demonstrates greatly elevated levels of GLUT1 as compared to cells transduced with an empty vector control (Figure 6A). Overexpression of GLUT1 increased ^3^H-2-DOG transport greater than five-fold as compared to the empty vector control cells (Figure 6B), demonstrating that the overexpressed GLUT1 was functional. The overexpression of GLUT1 in 85815GL cells did not increase proliferation and in fact proliferation slowed slightly by day 3 (Figure 6C).

### Overexpression of GLUT1 accelerates tumor formation

**Figure 7:**
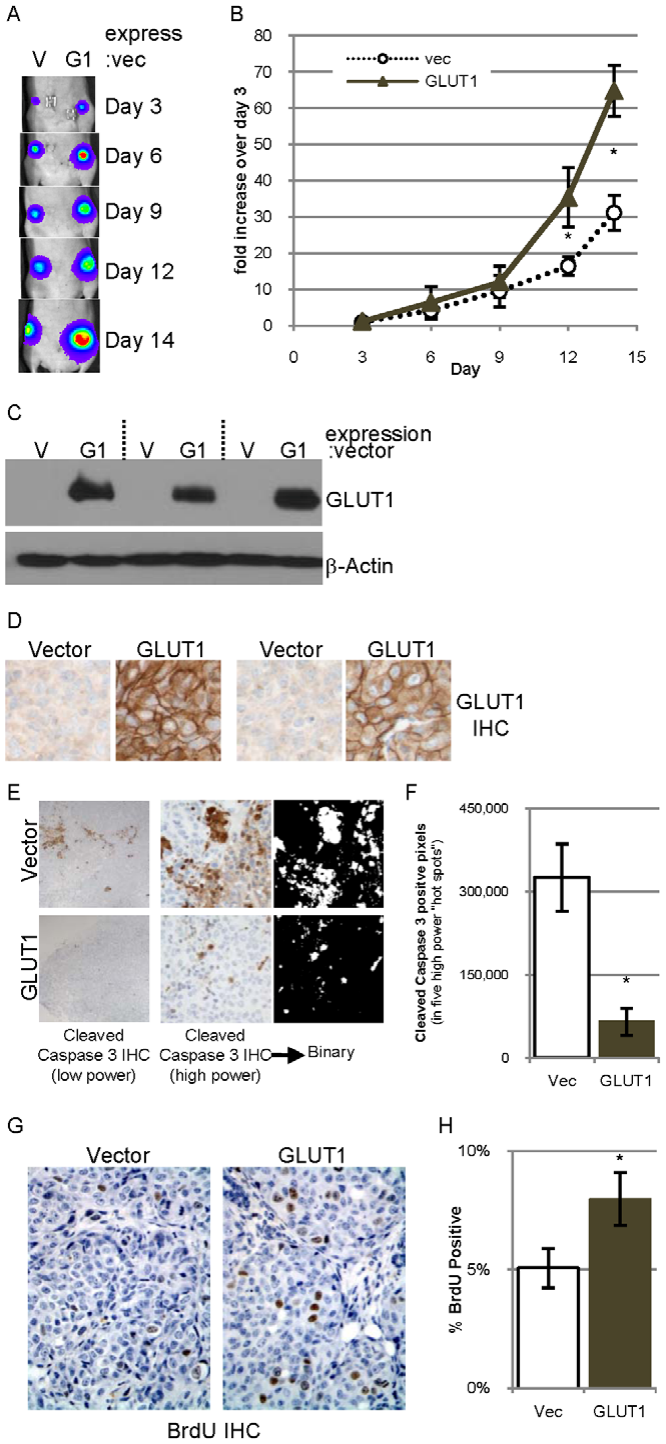
Overexpression of GLUT1 in 85815GL cells accelerates tumor formation. **A.** 0.4 million 85815GL cells expressing control vector (V) or GLUT1 (G1) were injected into contralateral #4 mammary fat pads of athymic nude mice. Bioluminescence from the labeled tumor cells was detected on days 3, 6, 9, 12 and 14 after implantation. **B.** The bioluminescence on days 3, 6, 9, 12 and 14 normalized to the day 3 value was averaged for all five mice and is presented +/−SEM. **C.** GLUT1 and β-actin expression evaluated in lysates of three tumor pairs by immunoblot analysis. **D.** GLUT1 expression evaluated by IHC in two tumor pairs. **E.** Low power photomicrographs of a pair of tumor sections derived from a vector control tumor (top) and a tumor overexpressing GLUT1 (bottom) immunostained for cleaved caspase 3 (left). High power photomicrographs of a pair of tumor sections immunostained for cleaved caspase 3 (middle), which are converted to binary pictures (right). **F.** Quantification of the number of cleaved caspase 3 positive pixels in five high power field “hot spots” in four tumors of each group. **G–H.** Representative high power photomicrographs of tumor sections immunostained for BrdU with hematoxylin counterstain (**G**) which is quantified (**H**).

To examine whether overexpression of GLUT1 stimulates tumor growth, 85815GL cells overexpressing GLUT1 or empty vector were injected into contralateral mammary glands of five athymic nude mice and tumor growth monitored by bioluminescence on days 3, 6, 9, 12 and 14 post tumor cell transplant (Figure 7A). Each tumor derived from cells overexpressing GLUT1 was bigger than the contralateral control tumor by day 14, and the average bioluminescence shows that as a group the tumors overexpressing GLUT1 were bigger on days 12 and 14 (Figure 7B). Additionally, weights of tumors harvested on day 14 demonstrate that all five tumors derived from tumors overexpressing GLUT1 are larger (by 6–73%) than the contralateral tumor (data not shown).

Immunoblot and immunohistochemistry analysis demonstrated that tumors derived from 85815GL cells overexpressing GLUT1 maintain robust expression of GLUT1 while the control tumors have very little GLUT1 expression (Figure 7C–D). Low power photomicrographs of cleaved caspase 3 stained sections demonstrate that tumors derived from 85815GL cells overexpressing GLUT1 appear to have less apoptosis than the control tumors (Figure 7E left), which is in contrast to the low levels of cleaved caspase 3 observed in the previous two models (Figures 3E and 5E). Indeed, tumors overexpressing GLUT1 have five times less cleaved caspase 3 staining than the control tumors (Figure 7F). Tumors derived from 85815GL cells overexpressing GLUT1 also have increased proliferation as determined by BrdU nuclear incorporation (Figure 7G–H). These data suggest that the increased tumor formation by 85815GL cells overexpressing GLUT1 results from both a decrease in apoptosis and an increase in proliferation.

## Discussion

We have demonstrated that GLUT1 is the most highly expressed hexose transporter in ErbB2- and PyVMT-induced mouse mammary carcinoma models, and that reducing the level of GLUT1 using shRNA or Cre/lox results in reduced glucose usage, reduced growth on plastic and in soft agar, and impaired tumor growth in nude mice. Cells with low levels of GLUT1 demonstrate reduced flux of glucose into lipid, while the ATP concentrations appear unaffected by impaired GLUT1 expression suggesting that reduced glucose consumption impairs lipid synthesis rather than ATP production. Overexpression of GLUT1 in a cell line that has very low levels of GLUT1 increases glucose transport and increases tumor growth (while cell proliferation is unaffected by GLUT1 overexpression *in vitro*). Together, this suggests that GLUT1 and glucose usage are important aspects in the tumor cell biology of breast cancer cells and impact both tumor growth and survival. This has also been suggested by groups who inhibited GLUT1 in cancer cells derived from other tissues, although most of this work focused on cell culture models [17], [36], [37].

Expression of GLUT1 is regulated by a number of mechanisms. Hypoxia induces the stabilization of the HIF transcription factors which induce expression of GLUT1 along with a panel of glycolysis genes [3]. In our study, we harvested our tumors when they were relatively small (∼200 mg), in an attempt to address GLUT1 function in tumors with minimal or no hypoxia. A number of cancer genes, including Myc [38], Akt [7], Ras [6], [39], Raf [39], Src [6], [40], EGFR [10], and loss of p53 [41], [42] induce increased glucose uptake which is associated with an increase in glycolysis. Some of these genes are known to promote localization of GLUT1 at plasma membrane [43], [44] or induce transcription of the GLUT1 gene [6], [39], [45], [46]. These diverse mechanisms by which cancer cells induce GLUT1 suggest that GLUT1 could be a central metabolic therapeutic target, which merits further pre-clinical investigation. Our data suggests that targeting GLUT1 alone may not suffice to kill tumor cells, but it may prove as a useful target in combination with therapies that target other molecules that regulate tumor metabolism. For example, inhibition of GLUT1 in combination with chemotherapeutic drugs cooperatively induced growth arrest and killed cancer cells in another model [37]. Therapies that when combined with targeted blockade of GLUT1 produce the best therapeutic index may be readily predicted based upon our current understanding of tumor metabolism since it is clear that tumors readily adapt to metabolic challenges. Thus unbiased approaches will be required to identify the best approaches to attack the altered metabolism present in tumors cells. It is also important to note that while the inhibition of glucose usage, lactate secretion and lipid synthesis appears to be modest in cells with reduced GLUT1 expression, these affects are similar to what has been observed in cells with inhibited PI3K/mTOR signaling by genetic or pharmacologic means [47], [48].

Two recent studies demonstrate interesting relationships between cancer oncogenes (KRAS, BRAF and ErbB2), regulation of glucose transport and cell survival under conditions of stress [39], [49]. Cancer cells with mutant KRAS or BRAF have higher levels of GLUT1 RNA and protein expression and higher rates of glucose uptake and glycolysis which allows the cells to survive in conditions of low glucose [39]. Cancer cells with wild type KRAS survived poorly in conditions of low glucose, but those that did survive upregulated GLUT1 and a small percentage acquired mutations in KRAS [39]. This data is compelling because it suggests that GLUT1 is an important stress-responsive mediator of cancer cell survival when glucose is limiting, making it an attractive therapeutic target. However, it also suggests that conditions of low glucose, which may be mimicked by reducing GLUT1 levels or function, may in fact select for mutations which would potentially lead to a more aggressive tumor. The second study demonstrated that mammary epithelial cells suffer a reduction in glucose transport and ATP production when they are detached from the substratum, but these deficiencies can be rescued by expression of ErbB2 [49]. This study links cell detachment, a condition necessary for migration/invasion, with sustained glucose transport and ErbB2 overexpression. This may be an important aspect to consider since the cells utilized in our study overexpress ErbB2 and maintained ATP levels when GLUT1 expression was reduced.

An alternative glycolytic pathway utilizing the M2 isoform of pyruvate kinase [50], [51] and identification of SGLT1 in EGFR expressing cancer cells [10] are recent discoveries in the field of cancer cell glucose metabolism which deviate from the dogmatic, textbook representation of glycolysis and suggest there is much still to learn about cancer cell metabolism. The use of the M2 isoform of pyruvate kinase by cancer cells is actually enzymatically inefficient, but promotes anabolic processes necessary for cancer cell growth and may in fact uncouple glycolysis from ATP production [51]. We observed decreased lipid synthesis in cells with reduced GLUT1 expression while ATP concentrations were unaltered, which suggests that this anabolic processes is affected by limiting GLUT1. Data demonstrating expression of the SGLT1 glucose transporter in tumor cells expressing EGFR [10] suggests that this second family glucose transporters merits examination in addition to the GLUT family. We were unable to detect SGLT1 RNA or protein expression in our systems, despite the fact that the EGFR was expressed in the mammary carcinoma cell lines. Glutamine and fatty acid metabolism are also critical for tumor cell proliferation [52], [53], [54], [55] and both of these carbon sources may replace or supplement the need for glucose; therefore these pathways must also be analyzed to gain a full picture of tumor cell metabolism. The metabolism of cancer cells is likely to be quite complex and appears to be quite adaptable in terms of carbon sources that can be utilized. The work presented here represents a single aspect of what is needed to fully understand cancer cell metabolism and how this understanding may impact the management or prognosis of affected patients.

## Supporting Information

Figure S1 **Colonies of 78617GL cells expressing control shRNA (shCTRL) or GLUT1 shRNA (shGLUT1) were grown in soft agar for three weeks (pictured in A) before being incubated 1 hr with 3 mg/ml BrdU then fixed in 10% neutral buffered formalin, embedded in paraffin, sectioned and stained by IHC for cleaved caspase 3 (B) or BrdU (C).**
(TIF)

Figure S2 **Schematic representation of the murine GLUT1 allele, the targeting vector and the recombinant allele. 5′ and 3′ probes that were used for Southern blot verification of homologous integration of the GLUT1 allele, and location of PCR primers that were used to additional verify 5′ integration is shown.**
(TIF)

Figure S3 **Schematic representation of wildtype, loxP-frt-Neo-frt-loxP, floxed GLUT1 allele, and deleted GLUT1 allele after Cre-mediated recombination are illustrated, along with a representative PCR analysis of the WT and loxP-frt-Neo-frt-loxP allele.** Location of primers used for genotyping are illustrated. PCR Primer sequences are: 2.85F – ctgtgagttcctgagaccctg; 2.9R – cccaggcaaggaagtagttc; FRTF – ctccattctccaaactaggaac; FRT-R2 – gaaggcacatatgaaacaatg.
(TIF)
