# Supplementary figures and images for: Modulation of Glucose Transporter 1 (GLUT1) Expression Levels Alters Mouse Mammary Tumor Cell Growth In Vitro and In Vivo
Source: PLoS One. 2011 Aug 3;6(8):e23205. doi: 10.1371/journal.pone.0023205 (PMC3149640; doi:10.1371/journal.pone.0023205)

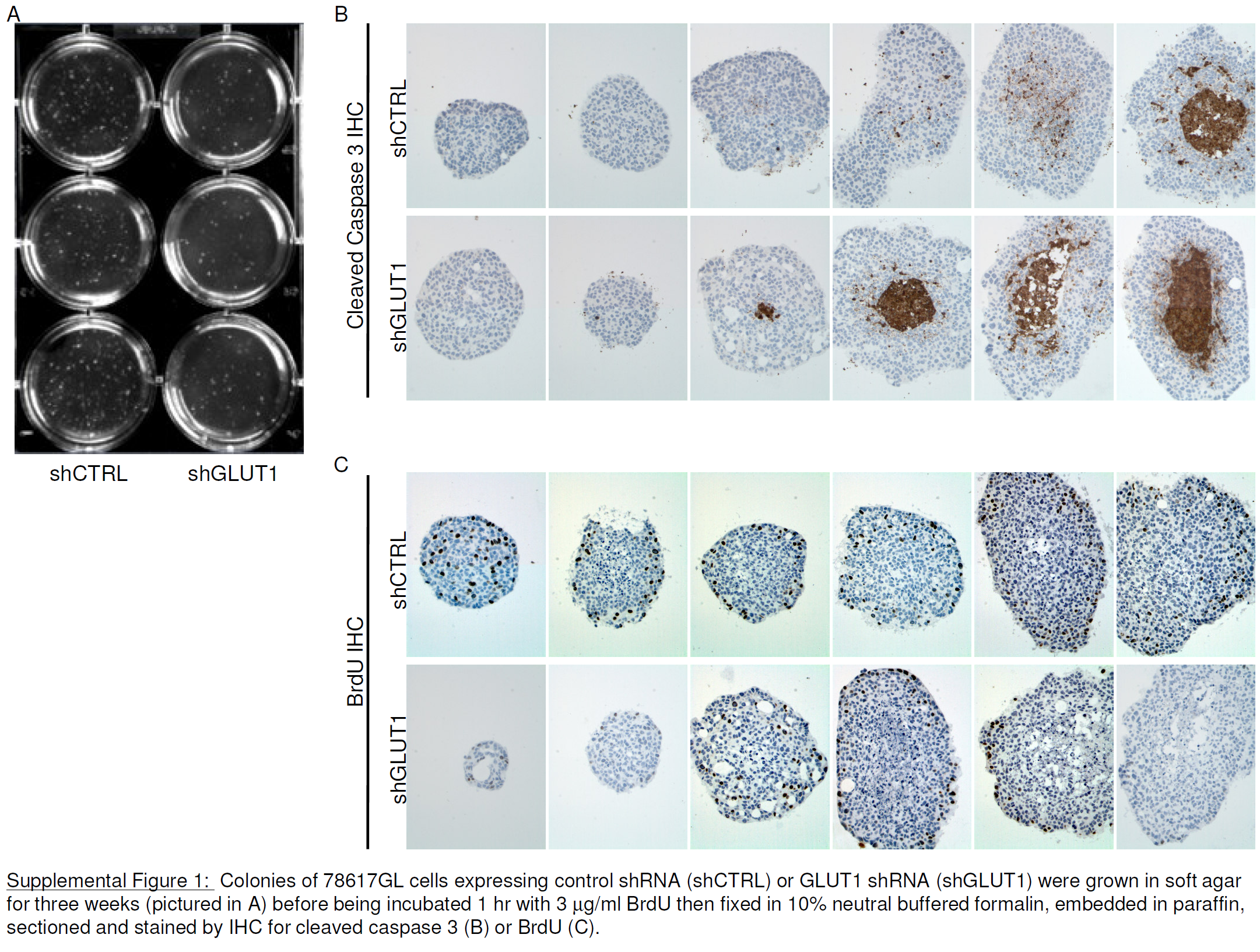

Supplement: Figure S1 — Colonies of 78617GL cells expressing control shRNA (shCTRL) or GLUT1 shRNA (shGLUT1) were grown in soft agar for three weeks (pictured in A) before being incubated 1 hr with 3 mg/ml BrdU then fixed in 10% neutral buffered formalin, embedded in paraffin, sectioned and stained by IHC for cleaved caspase 3 (B) or BrdU (C). (TIF) [file pone.0023205.s001.tif]

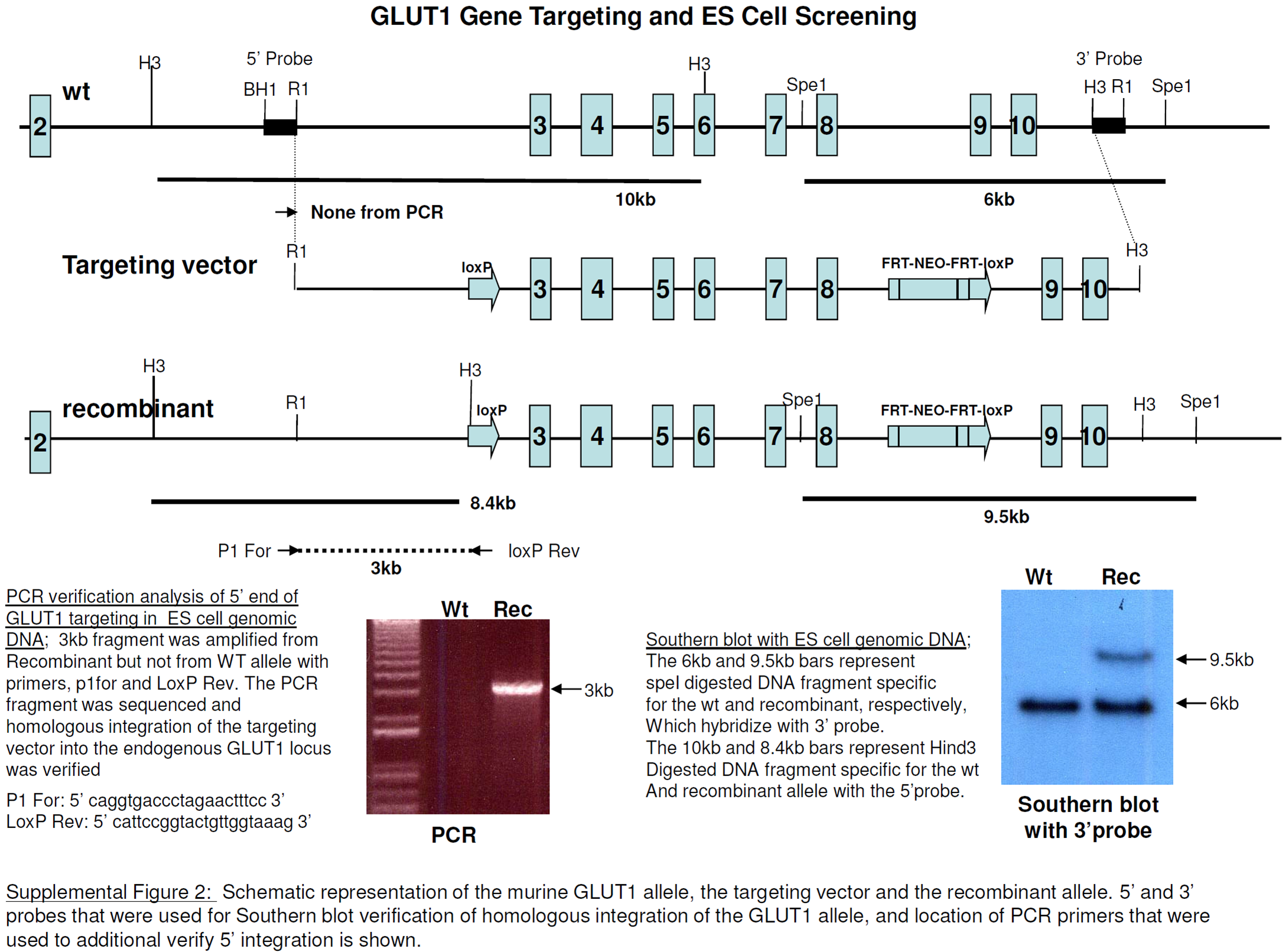

Supplement: Figure S2 — Schematic representation of the murine GLUT1 allele, the targeting vector and the recombinant allele. 5′ and 3′ probes that were used for Southern blot verification of homologous integration of the GLUT1 allele, and location of PCR primers that were used to additional verify 5′ integration is shown. (TIF) [file pone.0023205.s002.tif]

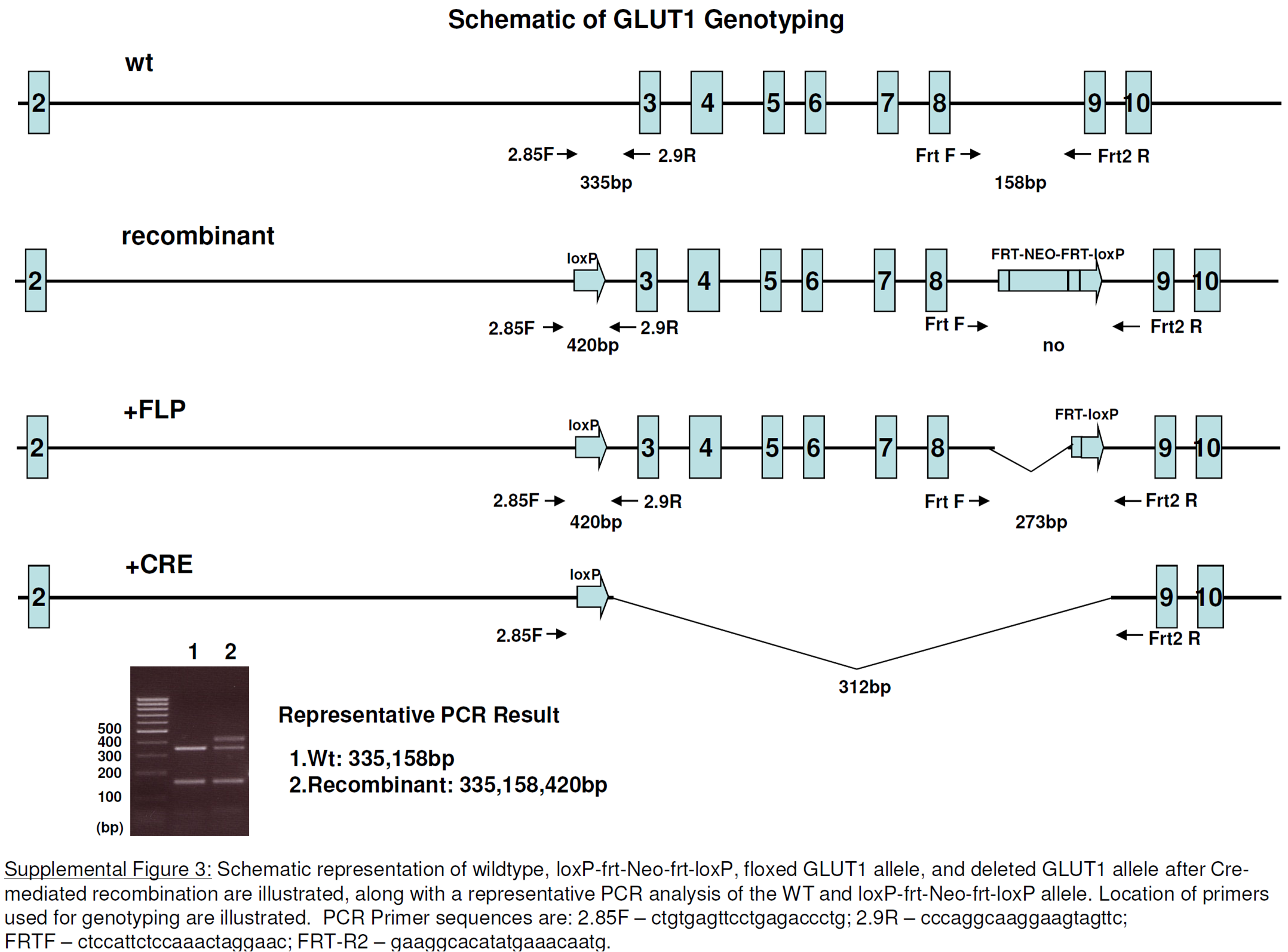

Supplement: Figure S3 — Schematic representation of wildtype, loxP-frt-Neo-frt-loxP, floxed GLUT1 allele, and deleted GLUT1 allele after Cre-mediated recombination are illustrated, along with a representative PCR analysis of the WT and loxP-frt-Neo-frt-loxP allele. Location of primers used for genotyping are illustrated. PCR Primer sequences are: 2.85F – ctgtgagttcctgagaccctg; 2.9R – cccaggcaaggaagtagttc; FRTF – ctccattctccaaactaggaac; FRT-R2 – gaaggcacatatgaaacaatg. (TIF) [file pone.0023205.s003.tif]
